# Comparative characterization of two GDP-mannose dehydrogenase genes from *Saccharina japonica* (Laminariales, Phaeophyceae)

**DOI:** 10.1186/s12870-016-0750-3

**Published:** 2016-03-08

**Authors:** Pengyan Zhang, Zhanru Shao, Weihua Jin, Delin Duan

**Affiliations:** Key Laboratory of Experimental Marine Biology, Institute of Oceanology, Chinese Academy of Sciences, Qingdao, 266071 China; Laboratory for Marine Biology and Biotechnology, Qingdao National Laboratory for Marine Science and Technology, Qingdao, 266071 China; University of Chinese Academy of Sciences, Beijing, 100049 China; State Key Laboratory of Seaweed Bioactive Substances, Qingdao, 266400 China

**Keywords:** *Saccharina japonica*, Alginate, GDP-mannose dehydrogenase, Enzyme kinetics, Stress response

## Abstract

**Background:**

*Saccharina japonica* is an important commercial brown seaweed, its main product is alginate, which is used in food, textile and by the cosmetic and pharmaceutical industries. GDP-mannose dehydrogenase (GMD) is the key enzyme involved in the synthesis of alginate. However, little is known about GMD in *S. japonica*. Here we report comparative biochemical analysis of two GMD genes in *S. japonica*.

**Results:**

Two GMD genes from *S. japonica* (*Sjgmd1*, *Sjgmd2*) were cloned. The open reading frame lengths of *Sjgmd1*, *Sjgmd2* are 963 bp and 948 bp, respectively. Alignment analysis showed that the two SjGMD sequences shared 79.38 % identity. Both proteins possess the GGxCLPKDV and GxGxVG sequence motifs characteristic of the short-chain dehydrogenase/reductase superfamily. The optimum temperatures for SjGMDs were 30 °C (SjGMD1) and 20 °C (SjGMD2), and the optimum pH values were 8.0 (SjGMD1) and 8.25 (SjGMD2). Kinetic analysis demonstrated the *K*m values for the substrate GDP-mannose were 289 μM (SjGMD1) and 177 μM (SjGMD2), and the *K*m values for the cofactor NAD^+^ were 139 μM (SjGMD1) and 195 μM (SjGMD2). The metal iron Zn^2+^ is a potent inhibitor of SjGMD1 and SjGMD2. Real-time PCR analysis showed that heat and desiccation treatments resulted in a significant increase in *Sjgmd1* and *Sjgmd2* transcript abundance, suggesting that the SjGMDs are directly involved in the acclimitisation of *S. japonica* to abiotic stresses.

**Conclusion:**

Our work identified two novel genes encoding GMD in *S. japonica*, comparatively characterized their structural characteristics and enzyme kinetics, and revealed the function of GMD in the stress adaptability of *S. japonica*. The knowledge obtained here enriched our understanding of the alginate synthesis mechanism in *S. japonica*, and may promote further research on functional differences between GMD genes.

**Electronic supplementary material:**

The online version of this article (doi:10.1186/s12870-016-0750-3) contains supplementary material, which is available to authorized users.

## Background

*Saccharina japonica* is one of the most important commercial brown seaweeds with an aquaculture production of about 5 million tons (wet weight) annually (http://www.fao.org/fishery/statistics/en). Besides serving as food, *S. japonica* is also used for alginate production [1]. Presently nearly 30,000 tons of algin are produced annually [2].

Alginate (C_6_H_8_O_6_)_n_ is a β-1, 4-linked linear heteropolymer consisting of variable amounts of β-D-mannuronic acid (M) and its C5-epimer α-L-guluronic acid (G) [3]. In the presence of divalent/trivalent cations, such as calcium, alginate can form algin, and its chemical properties vary with molecular weight and G/M ratio. Algin abundance and properties are different for different species [4], seasons [5], and environment conditions [1, 6, 7]. Algin possesses special gelling, viscosifying and stabilizing properties which gives it diverse applications in the food, textile, cosmetic and pharmaceutical industries.

Alginate synthetic pathways exist in bacteria (*Pseudomonas* and *Azotobacter*) and most marine brown seaweeds [8]. The synthetic pathway of bacterial alginate has been characterized in *A. vinelandii* and *P. aeruginosa* [9, 10]. The enzymes involved, AlgA [11], AlgC [12] and AlgD [13, 14], are responsible for the synthesis of the precursor GDP-mannuronic acid, Alg8 and Alg44 are involved in the polymerization of GDP-mannuronic acid [15, 16], and AlgG epimerizes the D-mannuronate residues into L-guluronate [2, 17]. The alginate synthetic mechanism is poorly understood in brown seaweeds. Lin et al. [18] initially proposed an alginate synthesis pathway for *Fucus gardner*, which was further verified in *Ectocarpus siliculosus* and *S. japonica* [19, 20]. Mannose-1-phosphate guanylyltransferase, phosphomannomutase, GDP-mannose 6-dehydrogenase (GMD), mannuronan synthase and mannuronate C5-epimerases (MC5E) have been identified in both *E. siliculosus* and *S. japonica* [19]. But so far, in brown algae, only MC5E genes from *L. digitata* and the GMD gene from *E. siliculosus* have been characterised biochemically [21, 22]. There is a need to carry out a functional analysis of the alginate synthetic genes in *S. japonica*.

Generally, GMD has been regarded as the rate-limiting enzyme in the alginate synthesis pathway [23, 24]. It catalyzes the synthesis of GDP-mannuronic acid with the reaction: GDP-Mannose + 2NAD^+^ + H_2_O → GDP-Mannuronic + 2NADH. In bacterial genomes, *gmd* is a single copy gene [25], while in the model brown alga *E. siliculosus*, it exists as a multi-copy gene, and only one *gmd* has been analyzed biochemically [22].

Based on our previous generated *S. japonica* transcriptome data [26], two GMD genes from *S. japonica* (*Sjgmd1* and *Sjgmd2*) were isolated and a functional analysis was conducted. It is expected to shed light on the structures of *Sjgmds* and their possible roles in algal adaptation to environmental stresses, to further enrich our understanding on the alginate synthesis in brown algae.

## Methods

### Sample collection

*S. japonica* “Zhong ke No. 2” were collected from cultivated rafts in Rongcheng, Shandong, China in 2014. Sampling permission was previously received from Shandong Gaolu aquatic product Co. Ltd.. Juvenile sporophytes (20 ~ 30 cm in length) in same habitat were selected as samples. The algal samples were washed with sterile seawater and precultured in darkness at 10 °C overnight. For the desiccation and heat-shock treatment, algal samples were desiccated in the darkness for 0 h, 0.5 h, 1 h, 1.5 h. Meanwhile, other algae were cultured in darkness at 25 °C for 0 h, 0.5 h, 1 h, 1.5 h, respectively. All the collected samples were frozen in liquid nitrogen and stored at −80 °C.

### Preparation of cDNA

Total RNA of *S. japonica* was extracted with RNeasy Plant Mini Kit (Qiagen, Germany) and quality was assessed using a DS-11 Spectrophotometer (Denovix, USA). High-purity RNA (OD_260_/_280_ = 1.8 ~ 2.2) were used for the synthesis of first strand cDNA according to the manual of the PrimeScript™ II 1st strand cDNA synthesis kit (Takara, Dalian, China). All templates were stored at −20 °C.

### Isolation of SjGMD genes

The candidate GMD unigenes were retrieved from our previous transcriptome database of *S. japonica* (GSE33853) [26], and identified by similarity analysis with the Blastx tool. To obtain the complete sequences of the GMD gene transcripts, 5′- rapid-amplification of cDNA ends (RACE) was conducted following the manual of the SMARTer RACE cDNA amplification kit (Clontech, USA), and 3′-RACE was performed using 3′-Full RACE Core Set Ver.2.0 (Takara, Dalian, China). All specific primers were designed by the Primer Premier 5 software (Table 1). Based on the assembled sequence information, the open reading frames (ORF) of *Sjgmd1* and *Sjgmd2* were amplified with two pairs of primers (GMD1-F/GMD1-R and GMD2-F/GMD2-R) (Table 1). PrimeSTAR max DNA polymerase was used in the PCR reaction, and the amplification program was as follows: 98 °C for 5 min, 35 cycles of 98 °C for 10 s, 55 °C for 5 s, 72 °C for 30 s, and 72 °C for 10 min.

**Table 1:** List of primers used in this study

Primer	Sequence (5′ to 3′)	Product size (bp)	Description
GMD1-3O	TGGTCCACAACTACGCCAACGGGCTCAAG	730	3′-RACE for *Sjgmd1*
GMD1-3I	CGACGCTCAGAAGGTGCT		
GMD2-3O	TTCCACCCGGACGACTGCCCGTCGGAC	491	3′-RACE for *Sjgmd2*
GMD2-3I	CGACACCCGGGAGCGCATGTCCAAC		
GMD1-5O	GGGACTGCTCAACGAGCTTCAGCACC	352	5′-RACE for *Sjgmd1*
GMD1-5I	GGCACCGAGAGGAGCACAATCTCAGG		
GMD1-F	CATATGATGGCTGAGGTAATGCCCAAGGAGAGC	963	ORF-PCR for *Sjgmd1*
GMD1-R	GAATTCTTAGGTGGTGAGCGAGTCGGGCGA		
GMD2-F	CATATGATGGCCGAGCCCGAGGTGAAGAAGT	948	ORF-PCR for *Sjgmd2*
GMD2-R	GAATTCTTAGATGATAAGCTCGCTCGGCGAC		
qGMD1-F	TCCTCTCACTCTTTCGGCATCC	128	qRT-PCR for *Sjgmd1*
qGMD1-R	CACCCGATCTGGATGATGCTC		
qGMD2-F	TGCTACCTGAGCCGCAAATACG	126	qRT-PCR for *Sjgmd2*
qGMD2-R	CCGCCAAGAACTCCCTGAAGACC		
Actin-F	GACGGGTAAGGAAGAACGG	184	qRT-PCR for *β-actin*
Actin-R	GGGACAACCAAAACAAGGGCAGGAT		

### Bioinformatic analysis of the SjGMD genes

Partial sequences of *Sjgmd* were assembled using DNAman 6.0 and the ORF was identified using the ORF finder tool (http://www.ncbi.nlm.nih.gov/gorf/gorf.html). The protein molecular weight (MW) and theoretical isoelectric point (pI) were predicted by the ProtParam [27], and the secondary structure of SjGMD1 and SjGMD2 were predicted with the SOPMA program [28]. A phylogenetic tree was constructed using the neighbor-joining algorithm in MEGA 6.0 with 1,000 bootstrap replicates [29].

### In vitro expression and purification of SjGMD1 and SjGMD2

The pMAL system (New England Biolabs, USA) was used for expression and purification of fusion protein. Specific primers with *Nde*I and *EcoR*I digestion sites (GMD1-F/GMD1-R and GMD2-F/GMD2-R) were designed to amplify the ORF of *Sjgmd*. The purified amplification products were ligated into pMD-19T vector (Takara, Dalian, China) and digested with *Nde*I and *EcoR*I. The target bands were purified and recombined into the pMAL-c5X vector which was able to express maltose-binding protein (MBP) fusion proteins with a TEV protease cleavage site between the MBP and target protein. NEB express competent *Escherichia coli* were used to express recombinant protein, and positive clones were collected and used for expression analysis.

Various induction temperatures (15 °C, 25 °C, 37 °C) and isopropy-β-D-thiogalactoside (IPTG) concentrations (0.1 mM, 0.3 mM, 0.5 mM) were applied to optimize the expression of MBP-GMD. The cell pellet was resuspended in column buffer (20 mM Tris-HCl, pH 7.5, 200 mM NaCl, 1 mM ethylene diamine tetraacetic acid (EDTA), 1 mM NaN_3_, 1 mM DL-dithiothreitol (DTT), 200 μM NAD^+^, complete protease inhibitor) and the target protein was released from the cells by sonication and purified on an amylose resin column according to the instruction manual. The purified SjGMD1 and SjGMD2 proteins were concentrated with Amiconultra-15 centrifugal filter units (Millipore, MWCO 30 kDa) and verified using sodium dodecyl sulfate polyacrylamide gel electrophoresis (SDS-PAGE) gel (12 %).

### Enzyme assays

Enzyme assays were performed by monitoring change of OD_340_ absorption with the Powerwave HT microplate spectrophotometer (BioTek, USA). The enzymatic assay mixture (200 μL) contained 100 mM Tris-HCl buffer, 0.33 mM GDP-Mannose, 1 mM NAD^+^, and purified SjGMD protein (30 ~ 40 μg). The pH value of Tris-HCl buffer was adjusted corresponding to different reaction temperature. To optimize the reaction parameter, the catalytic detections were performed under different temperatures (20, 25, 27, 30, 32, 35 °C for SjGMD1, and 10, 15, 20, 25, 30 °C for SjGMD2) and different pH values (6.5, 7.0, 7.5, 8.0, 8.25, 8.5, 9.0, 9.5). Each reaction was carried out for 20 min. To calculate the *K*m of the SjGMDs, the catalytic rates were measured under various GDP-mannose or NAD^+^ concentrations (0, 1/60, 1/30, 1/15, 2/15, 4/15, 8/15 mM) under optimal conditions and the *K*m values were calculated by double-reciprocal plot. The effects of heavy metals were assessed by adding 1 mM MgCl_2_, MnCl_2_, CaCl_2_ and ZnCl_2_ to the standard reaction mixture. Each reaction was conducted with three replicates.

### GDP-mannuronic acid high performance liquid chromatography (HPLC) analysis

One volume of sodium phosphate buffer (100 mM, pH 3.0) was added to terminate the enzyme assay reaction [22]. Subsequently, the mixture was centrifuged and 20 μL of supernatant was injected into a HPLC system (Shimadzu-20A, Japan) with a Partisil 10 SAX column (250 × 4.6 mm, 10 μm particle size; Whatman, USA). 10 mM sodium phosphate (pH 3.0) and 750 mM sodium phosphate (pH 3.7) were used as the mobile phases A and B. The separation procedure was as follows: t_0_ min 3 % B; t_25_ min 40 % B; t_33_ min 75 % B; t_35_ min 75 % B; t_56_ min 3 % B with a flow rate of 1 mL min^−1^ at 30 °C. A ultraviolet spectrum (230 ~ 320 nm) was recorded using a Photo-Diode Array detector.

### Mass spectrometry (MS) analysis

After the removal of the enzyme from the reaction solution by centrifugation on an Amiconultra-15 centrifugal filter unit (Millipore, MWCO 10 kDa), the solution was separated on a Sephadex G-10 gel (1.0 cm × 20 cm) and eluted in water. Sugar was detected using the phenol-sulfuric acid method [30], and the fraction containing sugar was selected and lyophilized.

The powder was dissolved in CH_3_CN-H_2_O (1:1, v/v), and MS was performed on a LTQ ORBITRAR XL (Thermo Scientific, USA). Mass spectra were registered in the negative ion mode with a flow rate of 5 μL min^−1^. The optimized parameters were: capillary voltage, −3000 V; cone voltage −50 V; source temperature, 80 °C; dissolved temperature, 150 °C. The collision energy was between 10 and 50 eV. All spectra were analyzed by Xcalibur.

### Quantitative real-time PCR analysis of *Sjgmd1* and *Sjgmd2*

Two pairs of primers (qGMD1-F/qGMD1-R, and qGMD2-F/qGMD2-R; Table 1) were designed for the amplification of 128 bp and 126 bp fragments, respectively. Internal control tests were conducted with the specific primers Actin-F and Actin-R [26]. The real-time PCR was performed with the SYBR Premix Ex *Taq* II (Takara, Dalian, China) on a TP800 Thermal Cycler Dice Real Time System (Takara, Japan). The thermal cycling protocol was: 95 °C for 30 s, followed by 45 cycles of 95 °C for 5 s, 55 °C for 10 s and 72 °C for 20 s. Relative quantitative values were calculated by the 2^-∆∆Ct^ method [31], and statistical analysis was conducted with SPSS 19.0.

**Table 2:** Influence of metals on the enzyme activity of SjGMDs

Metals	Enzyme activity of SjGMD1	Enzyme activity of SjGMD2
	(% of control)	(% of control)
control	100.00 ± 15.76	100 ± 13.23
MgCl_2_	133.79 ± 16.33	120.25 ± 14.27
CaCl_2_	178.05 ± 10.10	71.86 ± 3.87
ZnCl_2_	0.75 ± 0.75	0.75 ± 0.24
MnCl_2_	94.01 ± 7.43	81.94 ± 2.18

## Results

### Cloning of *Sjgmd1* and *Sjgmd2*

Four candidate unigenes from transcriptome data (Unigene50429, Unigene52620, Unigene57613, and Unigene7396) were annotated as GMD genes. On this basis, primers were designed and 352-bp and 730-bp sequences were obtained by 5′-RACE and 3′-RACE amplifications for *Sjgmd1*. The full-length cDNA sequence of *Sjgmd1* (1523 bp, KP172530) was assembled and the length of the 5′-UTR, ORF and 3′-UTR were 84 bp, 963 bp and 476 bp, respectively. For *Sjgmd2*, a 491-bp sequence was obtained by 3′-RACE amplification and a full-length cDNA sequence (1083 bp, KP172531) was assembled, which contained the 5′-UTR (72 bp), the ORF (948 bp) and the 3′-UTR (63 bp).

### Alignment and structure analysis of SjGMDs

**Fig. 1:**
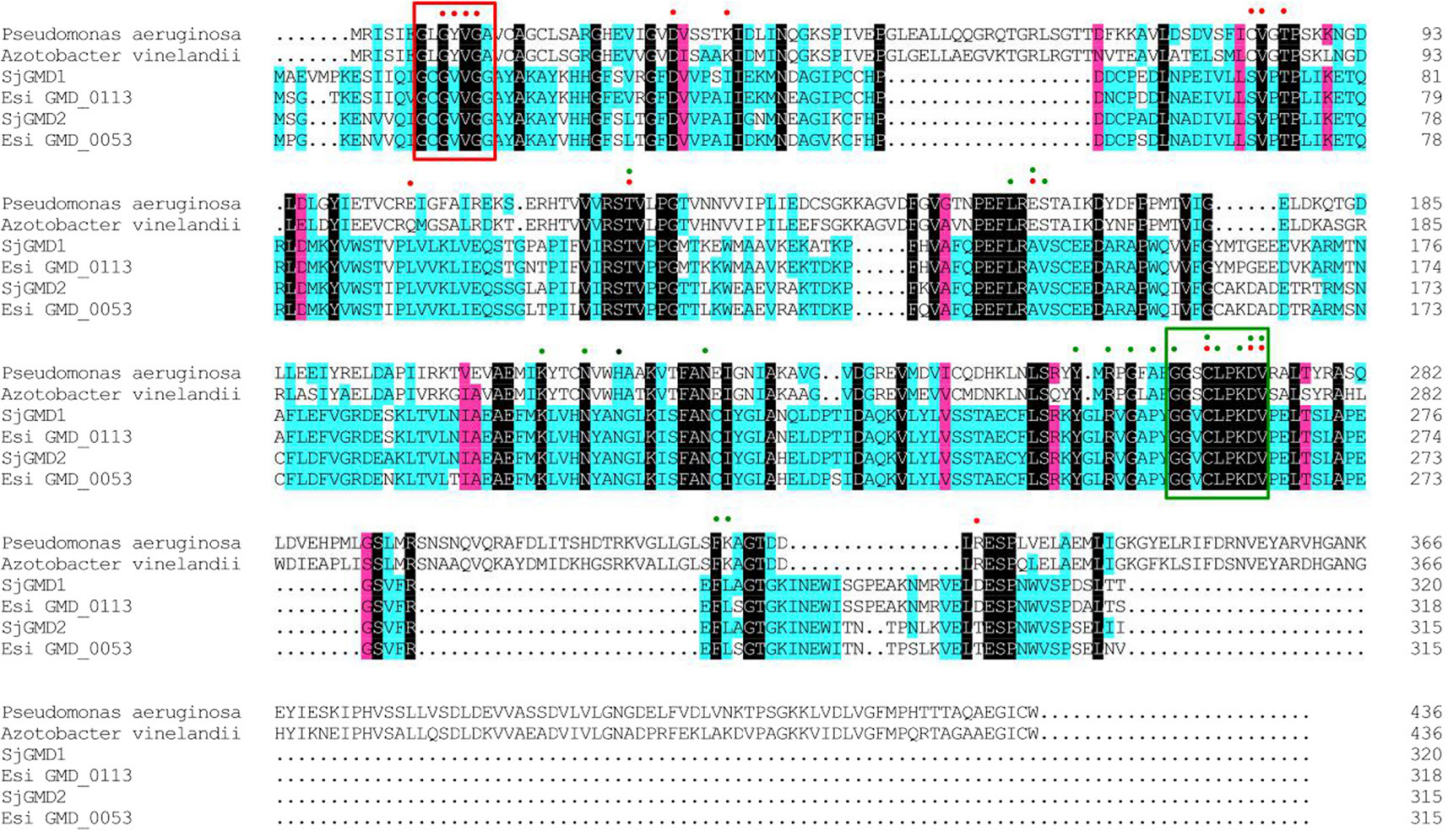
Multiple sequence alignment of SjGMDs with GMDs from other species. *Pseudomonas aeruginosa*, WP_034074438; *Azotobacter vinelandii*, AAB01487; *Esi* GMD_0113, CBJ27002; *Esi* GMD_0053, CBJ29903. The conserved NAD^+^-binding motif GxGxVG and the active motif GGxCLPKDV are represented with red and green frames, respectively; the candidate NAD^+^ and GDP-mannose binding sites of the SjGMDs are represented with red and green dots, respectively

*Sjgmd1* encoded a protein of 320 amino acids (35.20 kDa, pI 5.22) while *Sjgmd2* encoded a protein of 315 amino acids (34.46 kDa, pI 5.38). A homology analysis showed that the SjGMD proteins shared high similarity with members of the short chain dehydrogenase/reductase (SDR) superfamily. SjGMD1 shared 91.56 % identity with a GMD gene from *E. siliculosus* (CBJ27002), and SjGMD2 shared 93.97 % identity with another GMD gene from *E. siliculosus* (CBJ29903). However, the identity between SjGMD1 and SjGMD2 was only 79.38 % with most of the variance occurring outside the conserved catalytic region. A multiple sequence alignment of GMDs showed that both SjGMDs contained the conserved NAD^+^-binding motif GxGxVG (14 ~ 19 in SjGMD1 and 11 ~ 16 in SjGMD2) in the N-terminal region and the active motif GGxCLPKDV (259 ~ 267 in SjGMD1 and 256 ~ 264 in SjGMD2) in the C-terminal domain (Fig. 1). However, some key binding and catalytic residues differed between bacterial GMDs and SjGMDs, such as Val (17 in SjGMD1; 14 in SjGMD2), Ala (146 in SjGMD1; 143 in SjGMD2), Val (147 in SjGMD1; 144 in SjGMD2) and Pro (257 in SjGMD1; 254 in SjGMD2) (Fig. 1). Furthermore, as with the *E. siliculosus* GMDs [22] , SjGMDs contained incomplete N-terminal Rossmann folds. Predictions of secondary structure indicated that SjGMD1 contained 35.94 % random coil and 36.25 % α-helix, while SjGMD2 contained 35.24 % random coil and 33.33 % α-helix.

**Fig. 2:**
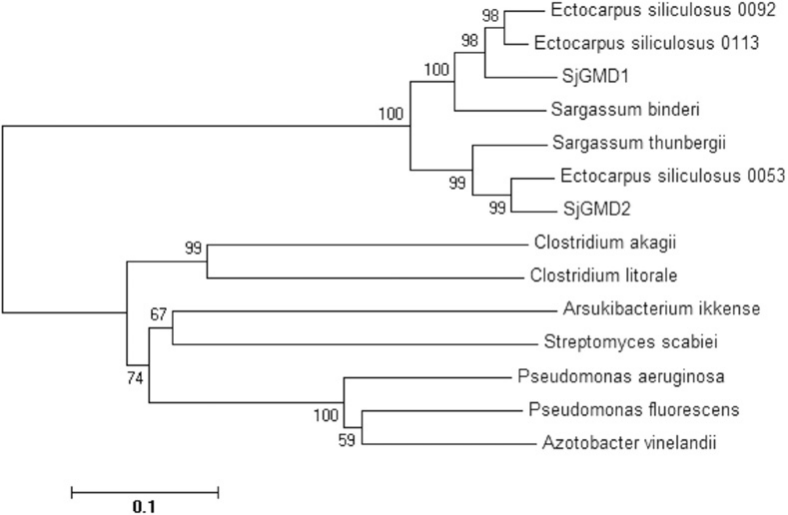
Phylogenetic tree constructed based on GMD sequences. The tree was constructed using the neighbor-joining algorithm with 1,000 bootstrap replicates. *Ectocarpus siliculosus* 0092, CBJ26993; *Ectocarpus siliculosus* 0113, CBJ27002; *Ectocarpus siliculosus* 0053, CBJ29903; *Sargassum binderi*, ESTs DV668856 + DV669914; *Sargassum thunbergii*, SRA073158; *Clostridium akagii*, WP_026882540; *Clostridium litorale*, WP_038266433; *Arsukibacterium ikkense*, WP_046556127; *Streptomyces scabiei*, WP_013000147; *Pseudomonas aeruginosa*, WP_034074438; *Pseudomonas fluorescens*, WP_042729895; *Azotobacter vinelandii*, AAB01487

To understand the evolutionary history of GMD, a phylogenetic tree was constructed. All the brown algal GMDs formed a single clade, while the bacterial GMDs clustered into a separate clade (Fig. 2). In addition, the brown algal clade tended to form two separate branches, each of which included one of the SjGMDs. SjGMD1 was closer to *E. siliculosus* 0113 (CBJ27002) and *E. siliculosus* 0092 (CBJ26993), while SjGMD2 was closer to *E. siliculosus* 0053 (CBJ29903).

### Expression of recombinant SjGMD1 and SjGMD2

MBP-SjGMD1 was successfully expressed after induction with 0.5 mM IPTG for 16 h at 25 °C, and its MW was consistent with the predicted MW of 77.7 kDa; while MBP-SjGMD2 was induced with 0.1 mM IPTG for 24 h at 15 °C, and its MW was consistent with the predicted MW of 76.96 kDa (Additional file 1). After being cleaved with factor Xa protease, the recombinant proteins were separated on SDS-PAGE (12 %), and had a MW of 35.2 kDa (SjGMD1) and 34.46 kDa (SjGMD2), respectively (Additional file 1). As no difference was detected between the activities of the fusion proteins and of the cleaved proteins, the recombinant SjGMD1 and SjGMD2 proteins were analysed directly in the enzymatic activity assay. The purified fusion proteins were concentrated to a final concentration of 3 ~ 4 mg mL^-1^ for the enzymatic activity assays.

### Enzymatic assay of SjGMD1 and SjGMD2 activities

**Fig. 3:**
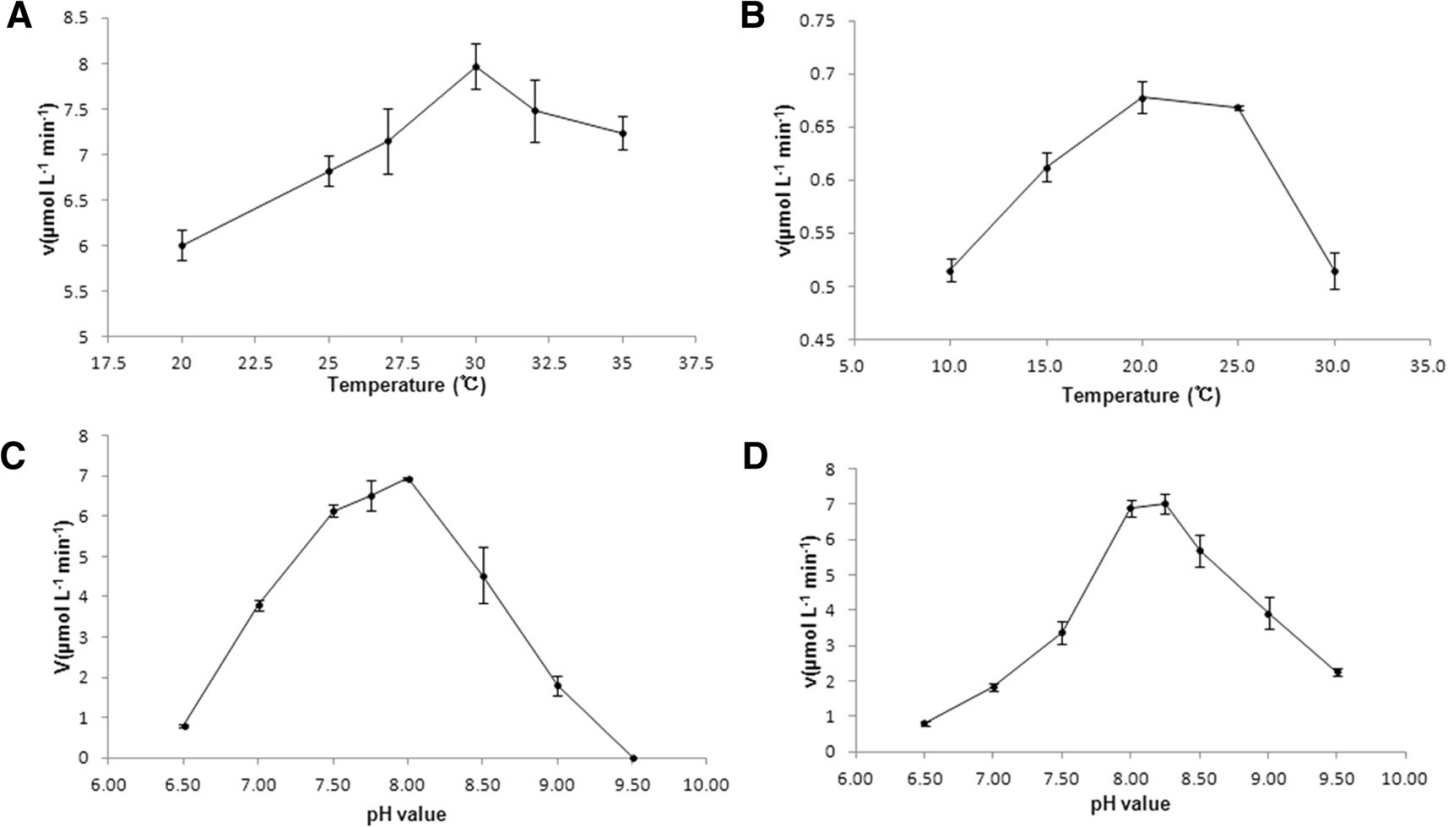
Influence of temperature and pH on the activity of SjGMDs. **a** influence of temperature (20 ~ 35 °C) on the activity of SjGMD1. **b** influence of temperature (10 ~ 30 °C) on the activity of SjGMD2. **c** influence of pH values (6.5 ~ 9.5) on the activity of SjGMD1. **d** influence of pH values (6.5 ~ 9.5) on the activity of SjGMD2

**Fig. 4:**
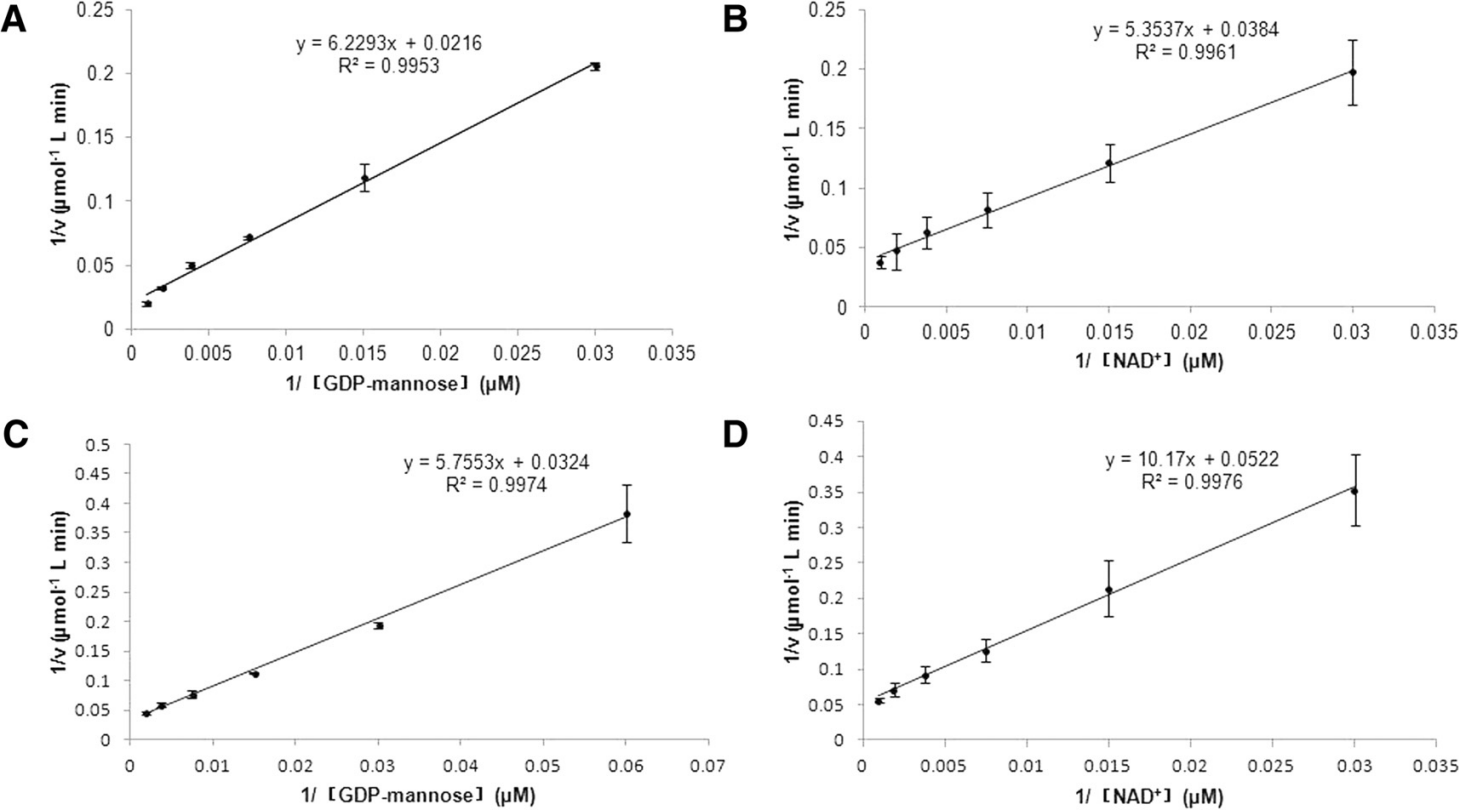
Kinetic analysis of SjGMDs. **a** the Lineweaver-Burk plots of SjGMD1 for the substrate GDP-mannose. **b** the Lineweaver-Burk plots of SjGMD1 for the cosubstrate NAD^+^. **c** the Lineweaver-Burk plots of SjGMD2 for the substrate GDP-mannose. **d** the Lineweaver-Burk plots of SjGMD2 for the cosubstrate NAD^+^. All the values represent means ± SD calculated from three reactions assays

The optimal temperatures were 30 °C (SjGMD1) (Fig. 3a) and 20 °C (SjGMD2) (Fig. 3b), and the optimal pH values were 8.0 (SjGMD1) (Fig. 3c) and 8.25 (SjGMD2) (Fig. 3d), respectively. Since these enzymes followed the typical Michaelis-Menten kinetics model, kinetic parameters were determined. The *K*m values of SjGMD1 were 289 μM for GDP-mannose (Fig. 4a) and 139 μM for NAD^+^ (Fig. 4b), while the *K*m values of SjGMD2 were 177 μM for GDP-mannose (Fig. 4c) and 195 μM for NAD^+^ (Fig. 4d). All the applied metal ions influenced the activity of SjGMDs, among which, ZnCl_2_ and MnCl_2_ were inhibitors of SjGMDs, while MgCl_2_ increased the activity of SjGMDs (Table 2). What’s more, CaCl_2_ acted as an activator of SjGMD1 but an inhibitor of SjGMD2 (Table 2).

### HPLC and MS analysis of GDP-mannuronic acid

**Fig. 5:**
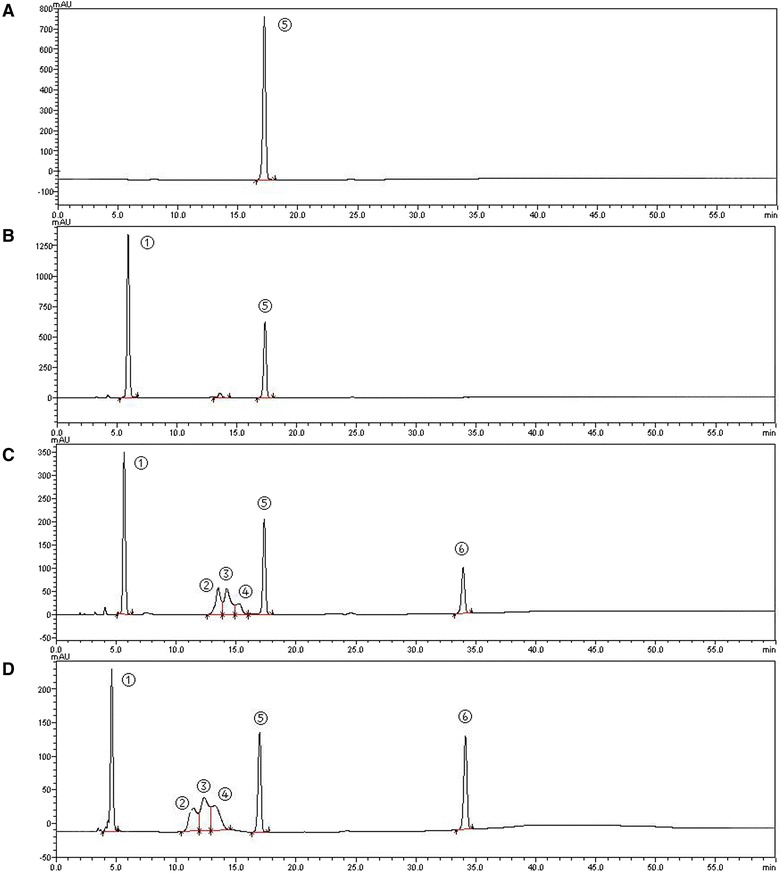
HPLC chromatography of SjGMD enzyme assays. **a** the separation of substrate GDP-Mannose; **b** ~ **d**, the separation of enzyme assay after 0, 5 and 30 min, respectively. ①, NAD^+^; ② and ④, unknown impurities from NAD^+^; ③, NADH; ⑤, GDP-Mannose; ⑥, GDP-Mannuronic acid

To confirm the catalytic activities of SjGMDs, HPLC analysis of the reaction solutions were conducted (Fig. 5). Figure 5a shows the peak of the substrate GDP-Mannose ⑤. Figure 5b shows that in the absence of GMD, only NAD^+^ ① and GDP-mannose ⑤ can be detected. In the following chromatograms which monitored products at reaction times of 5 min (Fig. 5c) and 30 min (Fig. 5d), the peak areas of NADH ③ and GDP-mannuronic acid ⑥ increased with the reaction time, while that of NAD^+^ ① and GDP-mannose ⑤ decreased significantly. This result demonstrated that GDP-mannuronic acid was oxidized from GDP-mannose, and both SjGMD1 and SjGMD2 were functional.

**Fig. 6:**
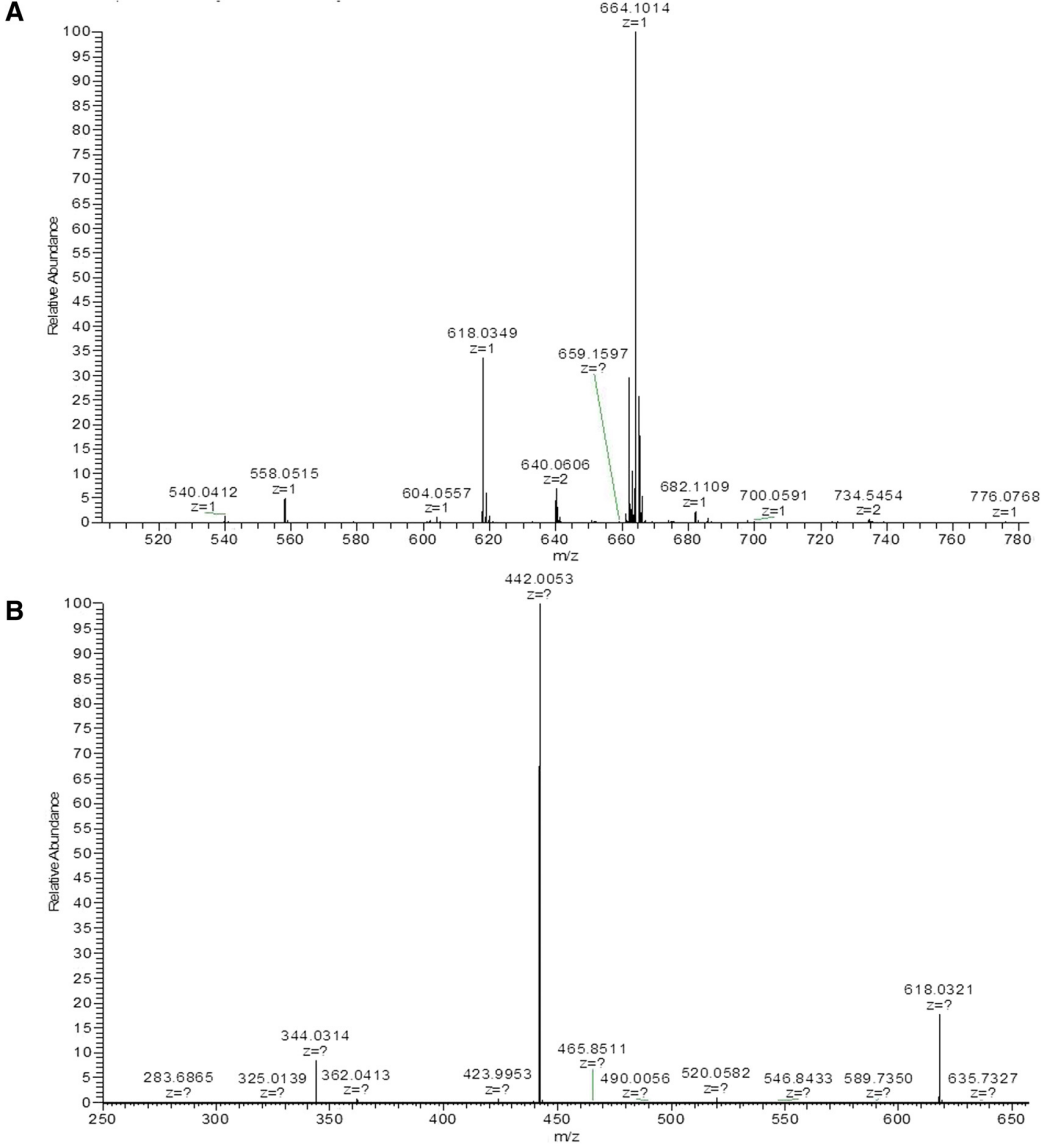
MS spectrum. **a** negative-ion mode ESI-MS spectrum of assay mixture in a range of 500 ~ 780. **b** negative-ion mode ESI-CID-MS/MS spectra of GDP-mannuronic acid (618.0349) in a mass range of 250 ~ 650

MS analysis showed that GDP-mannuronic acid and NADH could be easily identified from the high abundance trace, consistent with the predicted mass at 618 and 664 (Fig. 6a). In addition, collision induced dissociation (CID) fragmentation of the supposed GDP-mannuronic acid peak resulted in the loss of mannuronic acid or mannuronic acid without the phosphoric acid (Fig. 6b). These results further verified the production of GDP-mannuronic acid.

### Transcriptional analysis of the *Sjgmd*s

**Fig. 7:**
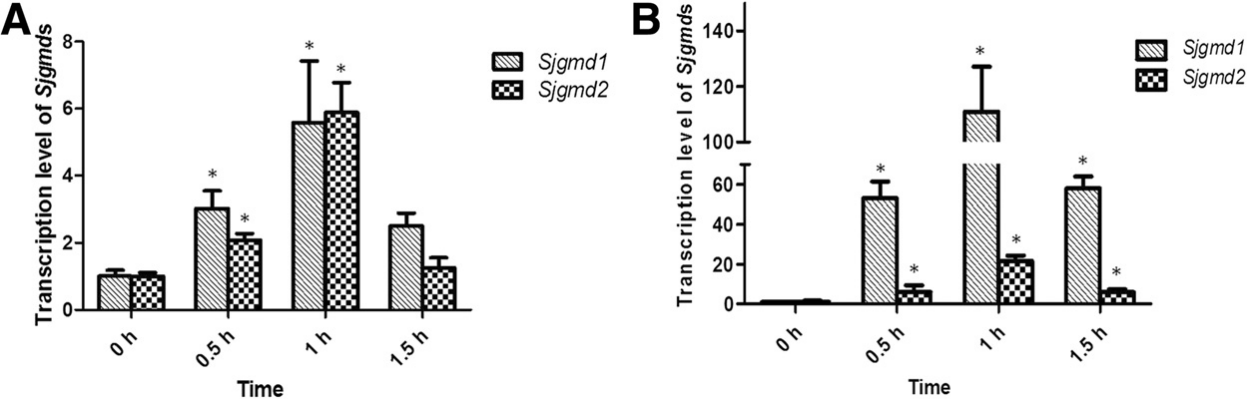
Influence of heat and desiccation treatments on SjGMD genes transcription level. **a** expression level of *Sjgmd*s under duration of heat stress. **b** expression level of *Sjgmd*s under duration of desiccation stress. The treatment groups which had significant difference (*P* < 0.05) with the controls were noted. All the data are the mean values of three independent experiments

Under heat and desiccation stresses, *Sjgmd1* and *Sjgmd2* exhibited the same transcriptional profiles (Fig. 7). After heat treatment, the level of *Sjgmd1* and *Sjgmd2* transcripts increased significantly (*P* < 0.05) and reached a peak after 1 h (5.52-fold increase for *Sjgmd1* and 5.86-fold increase for *Sjgmd2* compared with the control group), followed by an remarkable decrease to the original level at 1.5 h. A similar trend was detected under desiccation stress, the level of *Sjgmd1* and *Sjgmd2* transcripts reached a maximum after 1 h (110.63-fold increase for *Sjgmd1* and 19.94-fold increase for *Sjgmd2* compared with that of the control group).

## Discussion

Two novel GMD genes from *S. japonica* were isolated and characterized in this study. The alignment and phylogenetic analysis showed that the variance between the two GMDs in *S. japonica* is higher than that between *S. japonica* and *E. siliculosus*. This indicated that the two SjGMD genes might have envolved from different GMDs rather than being derived from a duplication event that happened in *S. japonica*. Previous research indicated that GMDs in brown algae had been gained by horizontal transfer of a single GMD from a bacterium [19, 20, 22]. Thus we proposed the GMD duplication event happened before the divergence of brown algae.

The crystal structure of GMD from *P. aeruginosa* has been reported and it showed that the enzyme contained two distinct domains connected by an R-helix and that both the N- and the C-terminal domains have a typical dinucleotide-binding Rossmann fold [14]. Although the characterized SjGMDs contained conserved motifs and residues, incomplete N-terminal Rossmann folds and residue substitutions were identified in SjGMDs, similar to the situation observed in *E. siliculosus.* These results suggested that brown algal GMDs may have a novel binding mechanism, suggesting distinct biochemical properties between GMDs in brown algae and in bacteria.

Though catalyzing the same reaction, the biochemical properties of SjGMD1 and SjGMD2 are quite different. The lower induction temperature indicated that SjGMD2 forms inclusion bodies more easily. The optimum temperature of SjGMD1 (30 °C) was the same as that of *E. siliculosus* GMD [22], while that of SjGMD2 (20 °C) was quite different from the GMDs of both *P. aeruginosa* (25, 37 or 50 °C) [32, 33] and *E. siliculosus* (30 °C). The optimal pHs (8.0; 8.5) of SjGMD1 and SjGMD2 were similar to those of *P. aeruginosa* (7.7) and *Arthrobacter* sp. (8.2) [13, 34]. This implied that the two SjGMDs exhibited different enzymatic activities. The *K*m of SjGMDs were much higher than those of bacterial GMDs [13], as which was found for *E. siliculosus* GMD. This may be explained by the fact that GDP-mannose is the common substrate of alginate and fucan synthesis, in that a lower *K*m might be beneficial for fucan formation [22]. Moreover, the activities of SiGMDs can be greatly affected by metal ions. Zn^2+^ was a strong inhibitor of both SjGMD1 and SjGMD2. This can be explained by its higher oxidability which might lead an oxidation of the catalytic residue Cys (262 in SjGMD1; 259 in SjGMD2), while Mg^2+^ can activate SjGMDs by improving the binding of substrate. More importantly, Ca^2+^ had contrasting effects on SjGMD1 and SjGMD2, which may be due to the structures of SjGMD1 and SjGMD2 undergoing different changes in configuration when bound to Ca^2+^. This suggested that, Ca^2+^ may act as a regulatory factor for SjGMD activities when the inside and outside environment of the cell change.

*S. japonica* is cold-temperate algal specie, distributed in the subtropical region of the northwest coast of the Pacific Ocean. Temperature and light are major factors that influence its growth. Desiccation can also threaten its survival. Under abiotic stresses, *S. japonica* can adjust its physiological and metabolic processes to acclimate and survive adverse conditions [35, 36]. As the first line of defense, the cell wall of brown algae can thicken to increase algal tolerance to stresses [37]. Alginate is the one of the major components of the brown algal cell wall, accounting for 45 % of the cell dry weight [38]. Thus, the increased alginate production might contribute to cell wall thickening. Under heat and desiccation stresses, the transcriptional level of *Sjgmd*s increased rapidly in a short time. This result indicated that *S. japonica* increases alginate synthesis by up-regulating the expression of genes involved in the synthetic pathway. SjGMD1 and SjGMD2 expression may contribute significantly to the adaptability of *S. japonica* in coastal environments, to assure normal growth of alga.

## Conclusions

In this article, two novel genes encoding GMD in *S. japonica* were cloned and verified. The SjGMDs exhibited sequence and structure differences and quite different biochemical properties and enzyme kinetics. The abundance of both *Sjgmds* transcripts increased under abiotic stresses, and this may contribute to a better adaptability of *S. japonica*. The knowledge obtained here enriched our understanding of alginate synthesis in brown algae, and provided a hint to study the functional differences between GMD genes.

## Availability of supporting data

The data sets supporting the results of this article are included within the article and its additional files.

## Additional file

Additional file 1: SDS-PAGE analysis of recombinant SjGMDs. (PDF 79 kb)

## Abbreviations

CID: Collision induced dissociation
DTT: DL-Dithiothreitol
EDTA: Ethylene diamine tetraacetic acid
G: α-L-guluronic acid
GMD: GDP-mannose dehydrogenase
HPLC: High performance liquid chromatography
IPTG: Isopropy-β-D-thiogalactoside
M: β-D-mannuronic acid
MBP: Maltose-binding protein
MC5E: Mannuronate C5-epimerases
MS: Mass spectrometry
MW: Protein molecular weight
ORF: The open reading frame
PI: Theoretical isoelectric point
RACE: Rapid-amplification of cDNA ends
SDR superfamily: Short-chain dehydrogenase/reductase superfamily
SDS-PAGE: Sodium dodecyl sulfate polyacrylamide gel electrophoresis
*Sjgmd*: GDP-mannose dehydrogenase gene from *S. japonica*
SjGMD: GDP-mannose dehydrogenase from *S. japonica*

## References

[1] Bertagnolli C, Espindola APDM, Kleinübing SJ, Tasic L, da Silva MG (2014). *Sargassum filipendula* alginate from Brazil: Seasonal influence and characteristics. Carbohyd Polym.

[2] Hay ID, Rehman ZU, Moradali MF, Wang YJ, Rehm BHA (2013). Microbial alginate production, modification and its applications. Microb Biotechnol.

[3] Evans LR, Linker A (1973). Production and characterization of the slime polysaccharide of *Pseudomonas aeruginosa*. J Bacteriol.

[4] Haug A, Larsen B, Baardseth E (1969). Comparison of the constitution of alginates from different sources. Proceedings of the VI International Seaweed Symposium.

[5] South GR (1979). Alginate levels in New Zeland *Durvillaea* (Phaeophyceae), withparticular reference to age variations in *D. antarctica*. Proceedings of the Interna-tional Seaweed Symposium.

[6] Larsen B, Salem DM, Sallam MA, Mishrikey MM, Beltagy AI (2003). Characterization of the alginates from algae harvested at the Egyptian Red Sea coast. Carbohyd Res.

[7] Davis T, Ramirez M, Mucci A, Larsen B (2004). Extraction, isolation and cadmium binding of alginate from *Sargassum* spp. J Appl Phycol.

[8] Draget KI, Moe ST, Skjåk-Bræk G, Smidsrød O, Stephen AM, Phillips GO, Williams PA (2006). Alginates. Food polysaccharides and their applications.

[9] Pindar DF, Bucke C (1975). The biosynthesis of alginic acid by *Azotobacter vinelandii*. J Biochem.

[10] Piggott NH, Sutherland IW, Jarman TR (1981). Enzymes involved in the biosynthesis of alginate by *Pseudomonas aeruginosa*. European J Appl Microbiol Biotechnol.

[11] May TB, Shinabarger D, Boyd A, Chakrabarty AM (1994). Identification of amino acid residues involved in the activity of phosphomannose isomerase-guanosine 5′-diphospho-D-mannose pyrophosphorylase. A bifunctional enzyme in the alginate biosynthetic pathway of *Pseudomonas aeruginosa*. J Biol Chem.

[12] Olvera C, Goldberg JB, Sánchez R, Soberón‐Chávez G (1999). The *Pseudomonas aeruginosa* algC gene product participates in rhamnolipid biosynthesis. FEMS Microbiol Lett.

[13] Roychoudhury S, May T, Gill J, Singh S, Feingold D, Chakrabarty A (1989). Purification and characterization of guanosine diphospho-D-mannose dehydrogenase. A key enzyme in the biosynthesis of alginate by *Pseudomonas aeruginosa*. J Biol Chem.

[14] Snook CF, Tipton PA, Beamer LJ (2003). Crystal structure of GDP-mannose dehydrogenase: a key enzyme of alginate biosynthesis in *P. aeruginosa*. Biochemistry.

[15] Remminghorst U, Rehm BH (2006). Alg44, a unique protein required for alginate biosynthesis in *Pseudomonas aeruginosa*. FEBS Lett.

[16] Remminghorst U, Rehm BH (2006). In vitro alginate polymerization and the functional role of Alg8 in alginate production by *Pseudomonas aeruginosa*. Appl environ Microb.

[17] Morea A, Mathee K, Franklin MJ, Giacomini A, O'Regan M, Ohman DE (2001). Characterization of algG encoding C5-epimerase in the alginate biosynthetic gene cluster of *Pseudomonas fluorescens*. Gene.

[18] Lin T-Y, Hassid W (1966). Pathway of alginic acid synthesis in the marine brown alga, *Fucus gardneri* Silva. J Biol Chem.

[19] Michel G, Tonon T, Scornet D, Cock JM, Kloareg B (2010). The cell wall polysaccharide metabolism of the brown alga *Ectocarpus siliculosus*. Insights into the evolution of extracellular matrix polysaccharides in Eukaryotes. New phytol.

[20] Ye N, Zhang X, Miao M, Fan X, Zheng Y, Xu D (2015). *Saccharina* genomes provide novel insight into kelp biology. Nat Commun.

[21] Nyvall P, Corre E, Boisset C, Barbeyron T, Rousvoal S, Scornet D (2003). Characterization of mannuronan C-5-epimerase genes from the brown alga *Laminaria digitata*. Plant physiol.

[22] Tenhaken R, Voglas E, Cock JM, Neu V, Huber CG (2011). Characterization of GDP-mannose dehydrogenase from the brown alga *Ectocarpus siliculosus* providing the precursor for the alginate polymer. J Biol Chem.

[23] Deretic V, Gill JF, Chakrabartay AM (1987). Gene algD encoding GDP-mannose dehydrogenase is transcriptionally activated in mucoid *Pseudomonas aeruginosa*. J Bacteriol.

[24] Tatnell PJ, Russell NJ, Gacesa P (1994). GDP-mannose dehydrogenase is the key regulatory enzyme in alginate biosynthesis in *Pseudomonas aeruginosa*: evidence from metabolite studies. Microbiology.

[25] Chitnis CE, Ohman DE (1993). Genetic-analysis of the alginate biosynthetic gene-cluster of *Pseudomonas aeruginosa* shows evidence of an operonic structure. Mol Microbiol.

[26] Deng Y, Yao J, Wang X, Guo H, Duan D (2012). Transcriptome sequencing and comparative analysis of *Saccharina japonica* (Laminariales, Phaeophyceae) under blue light induction. PLoS One.

[27] Gasteiger E, Hoogland C, Gattiker A, Duvaud SE, Wilkins M, Appel R, Walker J (2005). Protein identification and analysis tools on the ExPASy server. The Proteomics Protocols Handbook.

[28] Geourjon C, Deléage G (1995). SOPMA: significant improvements in protein secondary structure prediction by consensus prediction from multiple alignments. Comput Appl Biosc.

[29] Tamura K, Stecher G, Peterson D, Filipski A, Kumar S (2013). MEGA6: Molecular evolutionary genetics analysis version 6.0. Mol Biol Evol.

[30] DuBois M, Gilles KA, Hamilton JK, Rebers PA, Smith F (1956). Colorimetric method for determination of sugars and related substances. Anal Chem.

[31] Schmittgen TD, Zakrajsek BA, Mills AG, Gorn V, Singer MJ, Reed MW (2000). Quantitative reverse transcription-polymerase chain reaction to study mRNA decay: comparison of endpoint and real-time methods. Anal Biochem.

[32] Kimmel JL, Tipton PA (2005). Inactivation of GDP-mannose dehydrogenase from *Pseudomonas aeruginosa* by penicillic acid identifies a critical active site loop. Arch Biochem Biophys.

[33] Li F, Yu J, Yang H, Wan Z, Bai D (2008). Effects of ambroxol on alginate of mature *Pseudomonas aeruginosa* biofilms. Curr Microbiol.

[34] Preiss J (1966). GDP-mannose dehydrogenase from *Arthrobacter*. Methods Enzymol.

[35] Liu F, Wang W, Sun X, Liang Z, Wang F (2014). RNA-Seq revealed complex response to heat stress on transcriptomic level in *Saccharina japonica* (Laminariales, Phaeophyta). J Appl Phycol.

[36] Liu F, Wang W, Sun X, Liang Z, Wang F (2015). Conserved and novel heat stress-responsive microRNAs were identified by deep sequencing in *Saccharina japonica* (Laminariales, Phaeophyta). Plant Cell Environ.

[37] Fogg GE, Rai L, Gaur J (2001). Algal adaptation to stress-some general remarks. Algal Adaptation to Environmental Stresses.

[38] Kloareg B, Demarty M, Mabeau S (1986). Polyanionic characteristics of purified sulfated homofucans from brown-algae. Int J Biol Macromol.

