# Supplementary material for: Comparative characterization of two GDP-mannose dehydrogenase genes from Saccharina japonica (Laminariales, Phaeophyceae)
Source: BMC Plant Biol. 2016 Mar 8;16:62. doi: 10.1186/s12870-016-0750-3 (PMC4782291; doi:10.1186/s12870-016-0750-3)
Supplement: Additional file 1: — SDS-PAGE analysis of recombinant SjGMDs. (PDF 79 kb) [file 12870_2016_750_MOESM1_ESM.pdf]

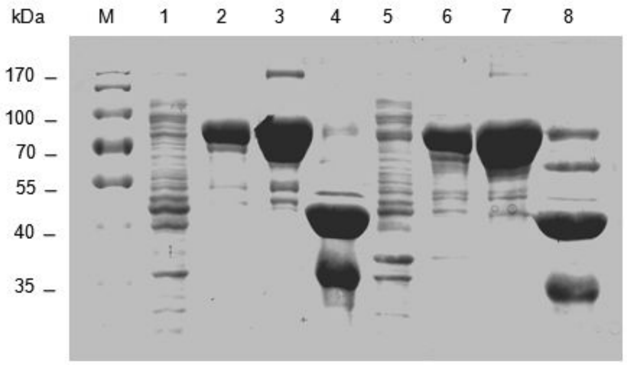

Additional file 1. SDS-PAGE analysis of recombinant SjGMDs. M: protein ladder; 1: non-induced expression of SjGMD1; 2: induced expression of SjGMD1 with 0.5 mM IPTG; 3: purified SjGMD1-MBP fusion protein; 4: SjGMD1 after factor Xa protease cleavage; 5: non-induced expression of SjGMD2; 6: induced expression of SjGMD2 with 0.1 mM IPTG; 7: purified SjGMD2-MBP fusion protein; 8: SjGMD2 after factor Xa protease cleavage.
